# Distribution of PD-L1, TROP2 and HER2- “lowness” in early triple-negative breast cancer: an opportunity for treatment de-escalation

**DOI:** 10.1007/s12094-023-03329-9

**Published:** 2023-10-18

**Authors:** Maria Jose Bueno, Silvana Mouron, Eduardo Caleiras, Mario Martínez, Luis Manso, Ramón Colomer, Miguel Quintela-Fandino

**Affiliations:** 1 Breast Cancer Clinical Research Unit, CNIO Spanish National Cancer Research Center, Melchor Fernandez Almagro 3, 28029 Madrid, Spain; 2 Histopathology Unit, CNIO Spanish National Cancer Research Center, Madrid, Spain; 3 Pathology Department, Hospital Universitario 12 de Octubre, Madrid, Spain; 4 Medical Oncology Department, Hospital Universitario 12 de Octubre, Madrid, Spain; 5 Medical Oncology Department, Hospital Universitario de La Princesa, Madrid, Spain

**Keywords:** Triple-negative breast cancer, PD-L1, TROP2, HER2-low, Carboplatin

## Abstract

**Background:**

HER2, TROP2 and PD-L1 are novel targets in triple-negative breast cancer (TNBC). The combined expression status of these targets, and whether they can define prognostic subgroups, is currently undefined.

**Methods:**

Immunohistochemistry was used to determine HER2, TROP2 and PD-L1 levels in 459 TNBC cases, that received in the adjuvant/neoadjuvant setting active surveillance, CMF, anthracycline-, anthracycline plus taxane-, or carboplatin-containing regimes.

**Results:**

HER2-low patients with PD-L1 > 1 CPS (double-positive, herein “DP”) had a mean PFS of 4768 days (95% CI: 4267–5268) versus 3522 days (95% CI: 3184–3861) for non-DP patients (*P* = 0.002). Regarding the received adjuvant treatment, DP patients (versus non-DP) receiving anthracyclines plus taxanes exhibited a mean PFS time of 4726 (95% CI: 4022–5430) versus 3302 (95% CI: 2818–3785) days (*P* = 0.039). Finally, 100% of DP patients that received a carboplatin-based regimen were long-term disease-free.

**Conclusions:**

Early HER2-low, PD-L1-positive TNBC patients have a very good prognosis, particularly if treated with anthracycline/taxane- or carboplatin-containing regimes.

## Introduction

Traditionally, triple-negative breast cancer (TNBC) has been viewed as a disease lacking druggable targets [1]. Recently, the positive effects of immunotherapies in the early [2, 3] and advanced [4–6] disease settings challenge this view. Similarly, the efficacy of the antibody–drug conjugates (ADCs) sacituzumab govitecan against TROP-2 and trastuzumab deruxtecan against HER2 have improved clinical outcomes in the general TNBC subpopulation [7] and in the HER2-low (positive, non-amplified) TNBC subpopulation [8], respectively. Whether these targets expression/co-expression and/or their levels of expression determine different TNBC sub-types, or drive its clinical history regardless of the use of those drugs is currently unknown; however, these datasets of the drug development focus on three novel targets (PD-L1, TROP2 and HER2) in this disease.

In the past, we have approached the complexity of TNBC with complex taxonomic techniques mixing genomics and phosphoproteomics to find prognostic [9] or predictive [10] factors in the early disease setting. This previous work was performed relying on a growing collection of TNBC samples (174 cases in the first study and 218 in the second). Now, we have expanded this collection to 459 TNBC cases and adopted a more pragmatic approach: while multi-platform (geno-proteomics), multi-marker-based classifications are unlikely to enter clinical routine practice soon due to technical, cost, and robustness or interpretation issues, histologic/immunohistochemistry markers are still the cornerstone of the routine decision tree in clinical routine breast cancer management. Since it is likely that these three targets are incorporated in the clinical routine soon, we wondered if the levels of these three new targets would condition the clinical course of TNBC cases not treated with drugs against them, and thus could be used to stratify TNBC cases to make clinical decisions; since our patient collection includes cases only up until 2015, none of them received (for the early disease setting management) any drug against those targets. The objective of this study was to determine the prognostic role of different combinations of PD-L1 and TROP-2 expression and HER2-low status in a cohort of early TNBC treated with chemotherapy only [cyclophosphamide plus methotrexate plus fluorouracil (CMF), anthracycline-, anthracycline plus taxane-, or carboplatin-containing regimes] or just active surveillance. Unexpectedly, we found that HER2-low, PD-L1-positive TNBC patients have an exceptional prognosis when treated with chemotherapy only, in particular when the treatment consisted on a carboplatin-based regime.

## Methods

This cohort was constituted by consecutive TNBC cases diagnosed in 3 hospitals of the Madrid area between January 2005 and December 2012. Patients with lack of metastatic disease at diagnosis were candidates for this study. A tissue micro-array was mounted using the primary tumor paraffin blocks. PD-L1 was determined with the Combined Positive Score (CPS) index (antibody 22C3 from DAKO); an H-score (0–3) was calculated for TROP2 (ABS380 from ENZO), whereas HER2-low status was determined by immunohistochemistry (4B5 from Ventana). Clinical data were collected using medical records. Relapse-free rates and hazard ratios were calculated with the Kaplan–Meier and Cox’s proportionate hazards methods, respectively. Two-sided *P* < 0.05 was considered significant for all tests. Data were analyzed with the SPSS Statistics Version 19 software. The study was approved by the Ethics Committee of the H12O (protocol approval #CEI 11/37); patients provided written informed consent. We followed the STROBE guidelines for cohort studies.

## Results

**Fig. 1:**
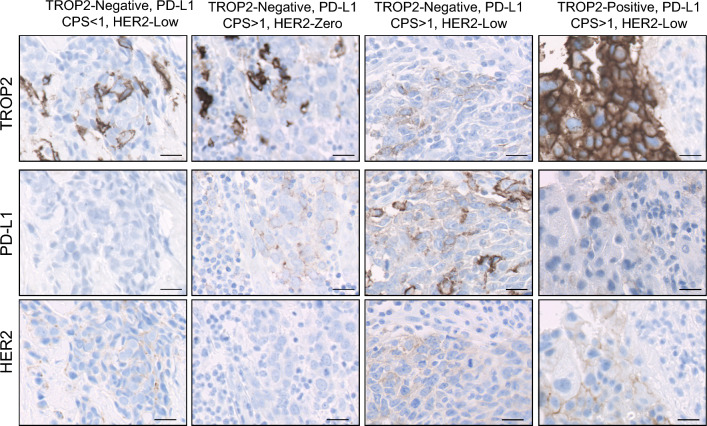
Staining examples and disease course according to PD-L1 or HER2-low status. Examples of four patients with different combinations of the three tested markers: TROP2-negative or-positive, PD-L1 CPS below or above 1, and HER2-low or HER2-zero

**Fig. 2:**
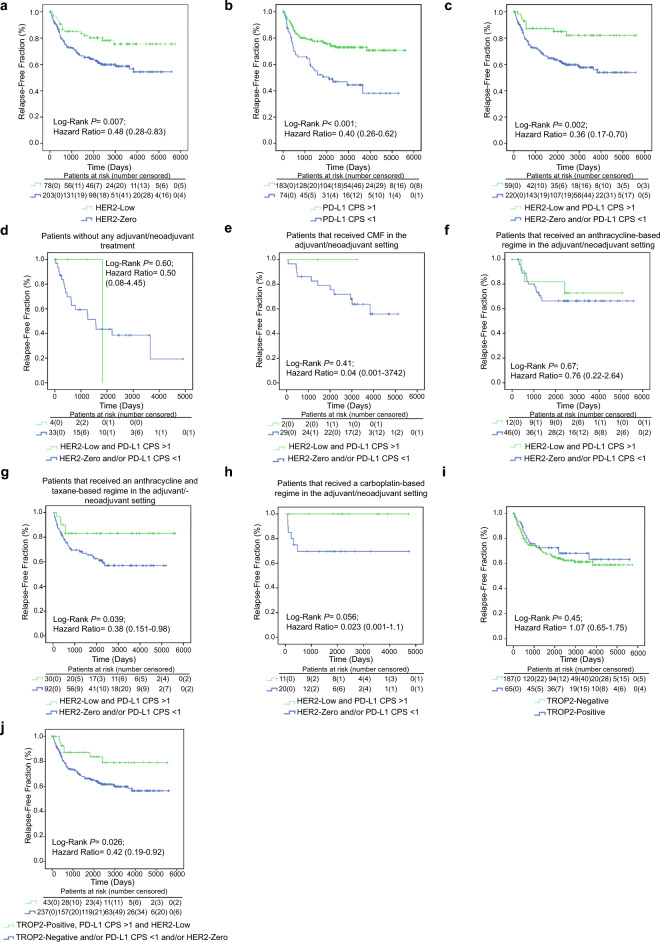
Disease course by type of chemotherapy (or active surveillance) in HER2-Low, PD-L1-positive patients (DP patients). **a** Relapse-free survival of HER2-Low versus HER2-Zero TNBC patients. **b** Same comparison for patients with PD-L1 CPS above (positive) or below (negative) 1. **c** DP patients versus non-DP patients (i.e., PD-L1>1 and HER2-Zero, PD-L1<1 and HER2-Low, or PD-L1<1 and HER2-Zero). **d** to **h** Comparison of the relapse-free times of DP versus non-DP patients when they received no adjuvant/neoadjuvant treatment (**d**), CMF (**e**), or an anthracycline-(**f**), anthracycline plus taxane-(**g**) or carboplatin-based (**h**) regime in the adjuvant/neoadjuvant setting, respectively. **i** Mean relapse-free survival of TROP2-Positive (3801 days) and TROP2-Negative (3967 days) patients. **j** When triple-positive patients (4619 days) were compared to non-triple-positive (3646 days) patients, relapse-free discrimination was not better than that for DP versus non-DP subgroups. 95% confidence intervals are included for hazard ratios

The cohort was constituted by 459 women with early TNBC; patients’ characteristics are depicted in Table 1. Median follow-up time was 2029 days. Twenty-seven point-five, 71.6 and 99.6% of the patients were HER2-low, had a PD-L1 CPS > 1 or displayed positive TROP2 staining, respectively (Fig. 1). A valid score for the three targets was available for 278 patients (60.5%). Of them, 57 (20.5%) patients were HER2-low and had a PD-L1 CPS score > 1 (“Double-positive”, herein DP). Clinical characteristics were not significantly different between patients according to the levels of each target or the DP status (Table 1). However, HER2-low, PD-L1-positive (CPS > 1) and DP patients had a significantly improved disease course (Fig. 2a, b and c, respectively). Regarding the HER2-low status, median relapse-free times were not reached in any of the two groups; the estimated mean relapse-free times were 4602 (95% CI 4109–5096) versus 3545 (95% CI 3192–3898) days for the HER2-low and HER2-zero subgroups, respectively (Fig. 2a). Concerning PD-L1 positivity, the estimated mean relapse-free times were 4257 (95% CI 3921–4593) versus 2714 (95% CI 2160–3268) days for the PD-L1-positive and -negative groups, respectively (Fig. 2b; medians not reached). Finally, the longest estimated mean relapse free was achieved by DP patients: 4768 days (95% CI 4267–5268); relapse-free time for non-DP patients was 3522 (95% CI 3184–3861) days (Fig. 2c; medians not reached).

**Table 1:** Clinical characteristics

	All patients (*N* = 459)	HER2-low (+ 1 or + 2) (*N* = 93)	PD-L1 CPS > 1 (*N* = 208)	TROP-2 positive^a^ (*N* = 194)	Double-posit.^+^ (*N* = 67)
Age (median, range)	54.0 (26.0–90.0)	57.1 (35.0–90.0)	54.0 (28.0–90.0)	54.9 (28.0–90.0)	58.4 (35–90)^#^
Tumor size					
T1	86 (18.7%)	21 (22.6%)	44 (21.2%)	41 (19%)	17 (25.4%)
T2	240 (52.3%)	55 (59.1%)	115 (55.3%)	124 (57.4%)	40 (59.7%)
T3	66 (14.4%)	11 (11.8%)	33 (15.9%)	29 (13.4%)	8 (11.9%)
T4	32 (7.0%)	4 (4.3%)	13 (6.3%)	16 (7.4%)	2 (3.0%)
N/A	35 (7.6%)	2 (2.2%)	3 (1.4%)	6 (2.8%)	0 (0%)
Nodal status					
N0	246 (53.6%)	53 (57.0%)	118 (56.7%)^#^	118 (54.6%)	38 (56.7%)
N1	96 (20.9%)	17 (18.3%)	46 (22.1%)	49 (22.7%)	13 (19.4%)
N2	49 (10.7%)	15 (16.1%)	30 (14.4%)	30 (13.9%)	13 (19.4%)
N3	30 (6.5%)	6 (6.5%)	10 (4.8%)	13 (6.0%)	3 (4.5%)
N/A	38 (8.3%)	2 (2.2%)	4 (1.9%)	6 (2.8%)	0 (0%)
Grade					
G1	8 (1.7%)	2 (2.2%)	3 (1.4%)	3 (1.4%)	2 (3%)
G2	88 (19.2%)	19 (20.4%)	37 (17.8%)	41 (19.0%)	12 (17.9%)
G3	291 (63.4%)	56 (60.2%)	142 (68.3%)	142 (65.7%)	44 (65.7%)
N/A	72 (15.7%)	16 (17.2%)	26 (12.5%)	30 (13.9%)	9 (13.4%)
Neoadjuvant/adjuvant/missing treatment (N, %)	147 (32.0%)/311 (67.8%)/1 (0.2%)	28 (30.1%)/64 (68.8%)/1 (1.1%)	71 (34.1%)/136 (65.4%)/1 (0.5%)	64 (29.6%)/152 (70.4%)/0 (0%)	24 (35.8%)/42 (62.7%)/1 (1.5%)
Type of chemotherapy					
None	53 (11.5%)	7 (7.5%)	19 (9.1%)	27 (12.5%)	4 (6.0%)
CMF	43 (9.4%)	5 (5.3%)	19 (9.1%)	23 (10.6%)	2 (3.0%)
Containing anthracyclines^1^	76 (16.6%)	18 (19.3%)	40 (19.2%)	39 (18.1%)	12 (17.9%)
Containing anthracyclines and taxanes^2^	177 (38,6%)	36 (38.7%)	83 (39.9%)	78 (36.1%)	30 (44.8%)
Containing carboplatin^3^	47 (10.2%)	12 (12.9%)	26 (12.5%)	23 (10.6%)	11 (16.4%)^#^
N/A	63 (13.7%)	15 (16.1%)	21 (10.1%)	26 (12.0%)	8 (11.9%)
HER2					
Zero	233 (50.8%)	N/A	134 (64%)	143 (62%)	N/A
Low	93 (20.2%)		67 (32.2%)	62 (28.7%)	
Amplified	4 (0.9%)^b^		0 (0%)	0 (0%)	
N/A	129 (28.1%)		7 (3.4%)	11 (5.1%)	
TROP2^a^					
Negative	72 (15.7%)	21 (22.6%)	50 (24.0%)	N/A	15 (22.4%)
Positive	216 (47.1%)	62 (66.7%)	146 (70.2%)		49 (73.1%)
N/A	171 (37.3%)	19 (10.8%)	12 (5.8%)		3 (4.5%)
PD-L1 CPS					
0	81 (17.6%)	17 (18.3%)	N/A	57 (26.4%)	N/A
> 1	208 (45.3%)	67 (72.0%)		146 (67.6%)	
N/A	170 (37.0%)	9 (9.7%)		13 (6.0%)	
Distant relapse					
No	265 (57.7%)	62 (66.6%)	137 (65.9%)	122 (56.5%)	50 (74.6%)
Yes	128 (27.9%)	16 (17.2%)	46 (22.1%)	65 (30.1%)	9 (13.4%)
N/A	66 (14.4%)	15 (16.2%)	25 (12%)	29 (13.4%)	8 (11.9%)

^1^AC, FEC or FAC

^2^AC followed by weekly paclitaxel, FEC followed by weekly paclitaxel, or TAC

^3^AC followed by weekly paclitaxel and carboplatin, or TC

^a^TROP2 positivity was defined by showing an H-score in quartiles 1 to 3 of the H-score distribution (cut-off point H-score = 1.48)

^b^Patients that were found to have an amplified HER2 were excluded from subsequent subgroup analysis

^+^Double-positive patients: patients whose tumors were HER2-low and had a PD-L1 CPS > 1

#Average age was older for DP patients compared with non-DP patients, with borderline statistical significance (*P* = 0.059). Nodal invasion distribution was statistically significantly different in PD-L1 positive versus negative patients (*P* = 0.048). In addition, treatment distribution was different in DP patients, with a higher proportion of patients receiving an anthracycline plus taxane- or carboplatin-based treatment-regime (*P* = 0.017). Of note, all parameters were compared among subgroups with a Chi-squared test, and the comparisons were-non adjusted for multiple-comparison testing. Thus, although after adjustment the treatment or nodal invasion distribution might have not been statistically significantly different among groups, the more conservative approach highlighting this potential imbalance was adopted

We then analyzed if the DP status on itself had prognostic (i.e., associated with better disease course in absence of treatment) or predictive (i.e., associated with benefit only in case of receiving certain chemotherapy regimes) value. To that end, we analyzed the PFS differences across the subgroups determined by the type of received adjuvant/neoadjuvant chemotherapy (or active surveillance; Table 1). Whereas for patients in the active surveillance group (Fig. 2d), CMF-treated (Fig. 2e) or anthracycline-treated (Fig. 2f) groups, we did not observe significant differences, DP status was associated with a more favorable disease course when patients received anthracyclines + taxanes or carboplatin-based regimes (Fig. 2g and h), with 100% of DP patients being relapse-free in the latter group. The relapse-free estimates for the anthracycline plus taxane-based regimes were 4726 (DP patients; 95% CI 4022–5430) and 3302 (non-DP patients; 95% CI 2818–3785) days; medians were not reached. In the carboplatin-based regime subgroup, because of the 100% progression-free rate, neither medians nor estimated mean PFS times could be calculated.

Determining TROP-2 levels did not refine risk stratification, neither when TROP-2 was considered alone or in combination with the positivity of HER2 and PD-L1 (Fig. 2i and j).

## Discussion

The recent improvements in early [2, 3] and advanced [4–8] TNBC are achieved at the expense of receiving up to 5-drug combos, which can cause significant permanent toxicity [11]. Current trials testing the addition of ADCs may add a sixth one; of note, T-DXD or sacituzumab govitecan is associated with high rates of grade 3–4 toxic events [7, 8], which may not be acceptable in the adjuvant setting. Whereas the determination of actionable targets is important for guiding drug development, it may be important as well for undertaking therapeutic decisions, particularly therapy de-escalation.

In this series, we report that early TNBC DP patients experience a favorable disease course when treated with chemotherapy alone. Of note, 100% of the patients are long-term disease free if treated with carboplatin-based regimens, an inexpensive option with predictable toxicity. Detecting a group with such a good long-term prognosis, that can be determine with inexpensive, easy and rapid techniques opens the opportunity to run de-escalation trials; clearly, the first external confirmation of our results should be in the Keynote 522 trial [2, 3], where we could obtain a preliminary evaluation of whether immunotherapy adds any further therapeutic benefit over a carboplatin-based combo alone. This could be followed by an additional randomized de-escalation trial for this patient population which represents 26% of the TNBC cases.

A question of key importance is which is the mechanism behind the increased sensitivity to platinum-based chemotherapy of the DP group, since understanding it may open avenues to turn sensitive as well the non-DP cases. First, observing the HR for disease relapse (Figs. 2a, b and c), it seems that PD-L1 positivity is a greater contributor to the protective effect of the “DP status” (HR = 0.40, compared to HR = 0.48 for HER2-lowness); however, interaction between the two factors should be explored in the future in independent, larger series. PD-L1 positivity has been correlated with TILs’ infiltration [although their co-occurrence seems to exert complex interactions [12], which, in turn, has been related as well to improved disease course in response to neoadjuvant chemo- or chemo-immunotherapy [13]]. However, in our series, we did not find that correlation, or a significant improvement in the prognosis accuracy of the model by adding TILs’ percentage to it (data not shown). An alternative explanation is that PD-L1 positivity may be an indirect marker of homologous recombination deficiency [14–17] (which, on the one hand, given the alterations in DNA repair, it may be linked to an increased mutational burden and neo-antigenic load, followed by PD-L1 expression as a compensatory mechanism, and, on the other hand, it is per se a sensitization factor for platinum-based therapies). The contribution of HER2-lowness is more intriguing. Although it has been reported that HER2-lowness is associated with a worse disease course in early hormone-positive breast cancer and a better disease course in TNBC [18], it has not been specifically associated with increased sensitivity to cisplatin. Future pre-clinical studies should address the interactions between PD-L1 and HER2 expression in DNA replication and/or its consequences in the STING-dependent immune response at the molecular level [19], as well as their impact in the tumor immune infiltrate.

The main limitation of our study is its retrospective nature and the relative low numbers in the subgroup analysis. The low numbers seem to affect, particularly, to the survival analysis in Fig. 2d and e, where the DP positive patients are less than 5 in both cases, an issue that does not happen, for example, in the analysis shown in 2 g or 2 h.

In conclusion, early HER2-low TNBC patients with a PD-L1 score of CPS > 1 have better prognosis compared to other TNBC patients, particularly when treated with carboplatin- or anthracycline plus taxane-containing regimes. HER2-low, PD-L1-positive status seems to be predictive of benefit from carboplatin-based treatment in the adjuvant setting.

## Data Availability

Anonymized data can be made available to any researcher upon request.
